# Evaluation of the Antimicrobial Activity of Some Components of the Essential Oils of Plants Used in the Traditional Medicine of the Tehuacán-Cuicatlán Valley, Puebla, México

**DOI:** 10.3390/antibiotics10030295

**Published:** 2021-03-12

**Authors:** Sebastián Candelaria-Dueñas, Rocío Serrano-Parrales, Marisol Ávila-Romero, Samuel Meraz-Martínez, Julieta Orozco-Martínez, José Guillermo Ávila-Acevedo, Ana María García-Bores, Carlos L. Cespedes-Acuña, Ignacio Peñalosa-Castro, Tzasna Hernandez-Delgado

**Affiliations:** 1 Laboratory of Natural Products Bioactivity, UBIPRO, Facultad de Estudios Superiores-Iztacala, Universidad Nacional Autónoma de México, Av. de los Barrios No. 1, Los Reyes Iztacala, Tlalnepantla 54090, Estado de México, Mexico; sebas5408@gmail.com (S.C.-D.); rocio.serrano@iztacala.unam.mx (R.S.-P.); avilaromero_mar@hotmail.com (M.Á.-R.); sammm@unam.mx (S.M.-M.); j.orozco@unam.mx (J.O.-M.); 2 Laboratory of Phytochemistry, UBIPRO, Facultad de Estudios Superiores-Iztacala, Universidad Nacional Autónoma de México, Av. de los Barrios No. 1, Los Reyes Iztacala, Tlalnepantla 54090, Estado de México, Mexico; tuncomaclovio@comunidad.unam.mx (J.G.A.-A.); boresana@iztacala.unam.mx (A.M.G.-B.); ipcastro@unam.mx (I.P.-C.); 3 Laboratorio de Fitoquímica-Ecológica, Grupo de Química y Biotecnología de Productos Naturales Bioactivos, Departamento de Ciencias Básicas, Facultad de Ciencias, Universidad del Bio Bio, Av. Andrés Bello s/n, P.O. Box 447, Ñuble, Chillán 3780000, Chile; ccespedes@ubiobio.cl

**Keywords:** antimicrobial activity, diterpenes, essential oils, monoterpenes, Tehuacán-Cuicatlán valley

## Abstract

In Tehuacán-Cuicatlán valley (Mexico), studies have been carried out on the essential oils of medicinal plants with antimicrobial activity and it was found that they present compounds in common such as: α-pinene, β-pinene, carvacrol, eugenol, limonene, myrcene, ocimene, cineole, methyl salicylate, farnesene, and thymol. The goal of this study was to assess the antimicrobial activity of essential oils’ compounds. The qualitative evaluation was carried out by the Kirby Baüer agar diffusion technique in Gram-positive bacteria (11 strains), Gram-negative bacteria (18 strains), and yeasts (8 strains). For the determination of the minimum inhibitory concentration (MIC), minimum bactericidal concentration (MBC), the agar dilution method was used. All the evaluated compounds presented antimicrobial activity. The compounds eugenol and carvacrol showed the largest inhibition zones. Regarding yeasts, the compounds ocimene, cineole, and farnesene did not show any activity. The compounds eugenol, carvacrol, and thymol presented the lowest MIC; bactericidal effect was observed at MIC level for *S. aureus* 75MR, *E. coli* 128 MR, and *C albicans* CUSI, for different compounds, eugenol, carvacrol, and thymol. Finally, this study shows that the essential oils of plants used by the population of Tehuacán-Cuicatlán valley share compounds and some of them have antibacterial and fungicidal activity.

## 1. Introduction 

One of the major health concerns today in the world is the emergence of new microbial strains resistant to the chemical substances used for their control [1]. During the last years, new strains resistant to commonly used antibiotics have appeared; for example, *Candida auris* is today an emergent and extremely dangerous strain difficult to control [2,3], as well as some other strains of *Enterobacter aerogenes, Escherichia coli, Pseudomonas aeruginosa, Staphylococcus aureus, Bacillus subtilis, Streptococcus pneumoniae,* and *Vibrio cholerae*, among others, causing serious health problems that are difficult to control and causing global concerns on the subject. 

On the other hand, the essential oils of plants have been developed as a commercial and pharmacological alternative for the treatment of various diseases, and their importance is based on the selective effects towards resistant strains that are recently reported. For example, it has been observed that clove oil, for which main component is eugenol, has antibacterial activity affecting respiratory metabolism, structural changes of DNA, and cell membrane permeability [4,5]. 

Therefore, it is important to carry out studies that validate the use of essential oils and to know the components that confer antimicrobial activity.

The International Union for the Conservation of Nature has decreed that Tehuacán-Cuicatlán valley [comprises the region from Puebla to Oaxaca State, México] is a center of mega diversity and endemism [6]. The valley is an area with high tradition in the use of medicinal plants [7]. Various studies have been carried out on the essential oils of some species such as: *Lantana achyranthifolia* Desf. (Verbenaceae) [8,9], *Lippia graveolens* H.B.K. (Verbenaceae) [9,10], *Cordia curassavica* (Boraginaceae) [11,12], and *Gymnolaena oaxacana* (Greenman) Rydb. (Asteraceae) [13], among others, and it was found that they contain many compounds in common among them: α-pinene, β-pinene, carvacrol, eugenol, limonene, myrcene, ocimene, cineole, methyl salicylate, farnesene, and thymol. Some studies have been carried out on these compounds, among which we can mention thymol [14,15], carvacrol [16,17], α-pinene [18], and eugenol [19,20,21] and it was observed that they present antimicrobial activity in both Gram-positive and Gram-negative strains affecting respiratory metabolism, structural changes of DNA, and cell membrane permeability.

In this way, this study aims to evaluate the antimicrobial activity of several compounds in the essential oils of plants used in the Mexican Traditional Medicine of the Valley of Tehuacán-Cuicatlán, Puebla, that include α-pinene, β-pinene, carvacrol, eugenol, limonene, myrcene, ocimene, cineole, methyl salicylate, farnesene, and thymol.

## 2. Results

### 2.1. Qualitative Evaluation of Antibacterial Activity

The results obtained in the evaluations of the antibacterial activity are shown in Table 1, Table 2 and Table 3.

As it can be seen, bacterial strains (Gram-positive and Gram-negative bacteria) and yeasts were sensitive to the tested compounds, and the ones that showed the highest inhibition zones for Gram-positive and Gram-negative bacteria were carvacrol and eugenol; α-pinene, β-pinene, and myrcene were the compounds that presented the lowest inhibition zones (Table 1 and Table 2). Regarding yeasts, the ocimene, cineole, and farnesene compounds did not show activity on any yeast strain (Table 3).

Considering the size of the inhibition zone with respect to the bacterial group (Gram-positive and Gram-negative) and yeast, an analysis of variance (ANOVA) was carried out. It can be observed that there are no significant differences (*p* < 0.05), i.e., the compounds inhibited the growth of both bacterial and yeast types.

### 2.2. Quantitative Evaluation

Once the antimicrobial activity of the different compounds had been verified, the minimum inhibitory concentrations (MIC) were determined. The results obtained are presented in Table 4, Table 5 and Table 6. 

The compounds carvacrol and eugenol presented the smallest minimum inhibitory concentrations for the bacterial strains (Table 4 and Table 5), i.e., low concentrations were required to drastically inhibit growth (0.03 to 0.75 mg /mL). The α-pinene and β-pinene compounds presented MICs above 4 mg /mL, these being the highest values for both bacterial types. The compounds carvacrol, eugenol, and thymol presented the lowest MICs in yeast (Table 6), and low concentrations of 0.062 to 0.25 mg/mL were required to drastically inhibit the growth of challenged populations. The other compounds that showed activity presented concentrations greater than or equal to 2 mg/mL.

The eugenol (0.125–0.250 mg/mL), carvacrol (0.03 mg/mL), and thymol (0.03–0.250 mg/mL) compounds were those that presented the lowest concentrations in yeasts (Table 6). 

As it can be seen in Table 4, Table 5 and Table 6, six of the tested compounds showed activity in multi-resistant strains (eugenol, limonene, carvacrol, methyl salicylate, farmesene, and thymol).

### 2.3. Time-Killing Curves

**Figure 1:**
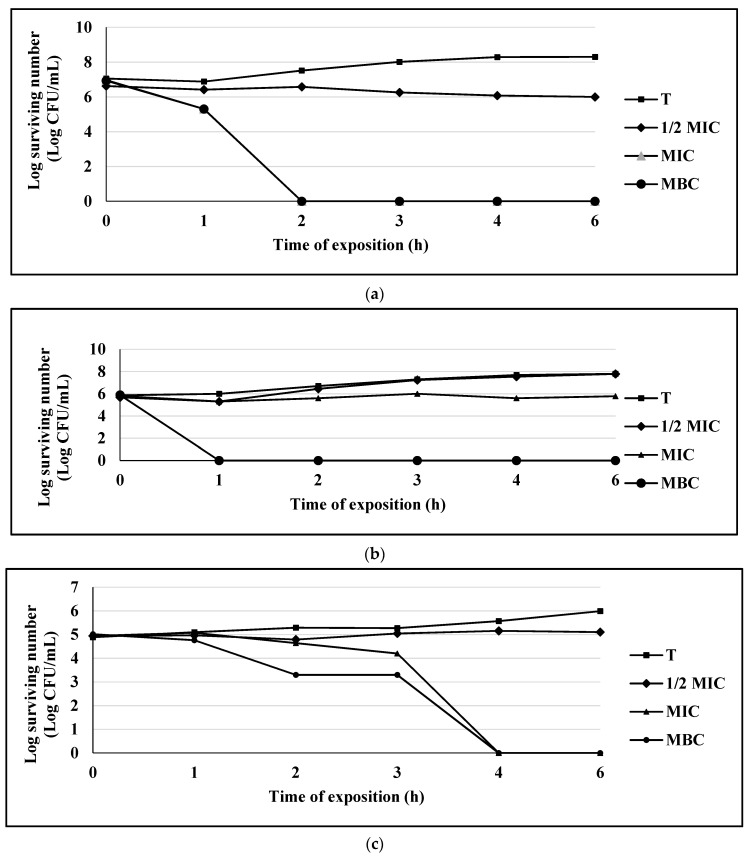
(**a**) Survival curve of *E. coli* exposed to eugenol. Data are represented as the mean ± S.E. (*n* = 3). The concentrations used were: 0.125 mg/mL (½ MIC), 0.25 mg/mL (MIC), and 0.5 mg/mL (MBC); the control group (T) only contains the bacterial culture. (**b**) Survival curve of *S. aureus* 75MR exposed to eugenol. Data are represented as the mean ± S.E. (*n* = 3). The concentrations used were: 0.25 mg/mL (½ MIC), 0.5 mg/mL (MIC), and 1.0 mg/mL (MBC); the control group (T) only contains the bacterial culture. (**c**) Survival curve of *C. albicans* CUSI exposed to eugenol. Data are represented as the mean ± S.E. (*n* = 3). The concentrations used were: 0.125 mg/mL (½ MIC), 0.25 mg/mL (MIC), and 0.5 mg/mL (MBC); the control group (T) only contains the bacterial culture.

**Figure 2:**
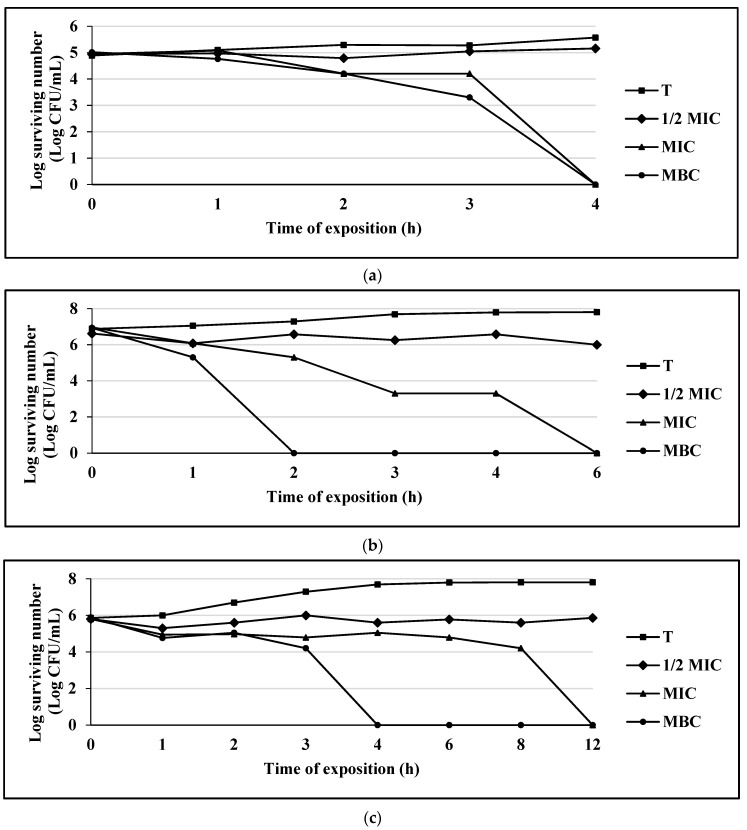
(**a**) Survival curve of *E. coli* exposed to carvacrol. Data are represented as the mean ± S.E. (*n* = 3). The concentrations used were: 0.062 mg/mL (½ MIC), 0.125 mg/mL (MIC), and 0.25 mg/mL (MBC); the control group (T) only contains the bacterial culture. (**b**) Survival curve of *S. aureus* 75MR exposed to carvacrol. Data are represented as the mean ± S.E. (*n* = 3). The concentrations used were: 0.062 mg/mL (½ MIC), 0.125 mg/mL (MIC), and 0.25 mg/mL (MBC); the control group (T) only contains the bacterial culture. (**c**) Survival curve of *C. albicans* CUSI exposed to carvacrol. Data are represented as the mean ± S.E. (*n* = 3). The concentrations used were: 0.01 mg/mL (½ MIC), 0.03 mg/mL (MIC), and 0.062 mg/mL (MBC); the control group (T) only contains the bacterial culture.

**Figure 3:**
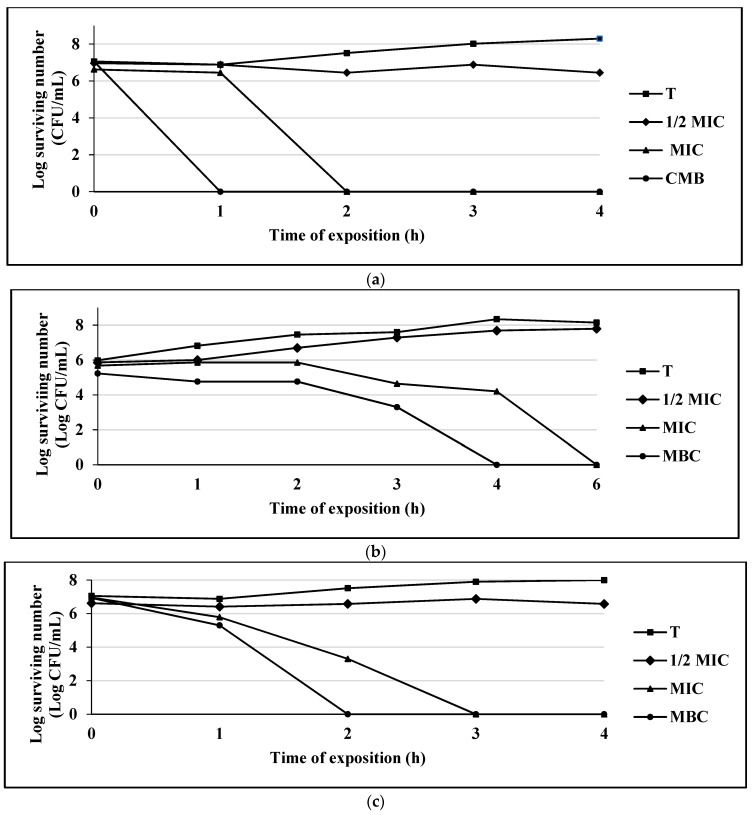
(**a**) Survival curve of *E. coli* exposed to thymol. Data are represented as the mean ± S.E. (*n* = 3). The concentrations used were: 0.125 mg/mL (½ MIC), 0.25 mg/mL (MIC), and 0.50 mg/mL (MBC); the control group (T) only contains the bacterial culture. (**b**) Survival curve of *S. aureus* 75MR exposed to thymol. Data are represented as the mean ± S.E. (*n* = 3). The concentrations used were: 0.25 mg/mL (½ MIC), 0.50 mg/mL (MIC), and 1.0 mg/mL (MBC); the control group (T) only contains the bacterial culture. (**c**) Survival curve of *C. albicans* CUSI exposed to thymol. Data are represented as the mean ± S.E. (*n* = 3). The concentrations used were: 0.01 mg/mL (½ MIC), 0.03 mg/mL (MIC), and 0.062 mg/mL (MBC); the control group (T) only contains the bacterial culture.

Once the MIC, MBC and MFC parameters were established, the effects of the compounds with the highest antimicrobial activity on a Gram-positive bacterium (*S. aureus* 75 MR), a Gram-negative bacterium (*E. coli* 128 MR) and a yeast (*C albicans* CUSI), for the compounds eugenol (Figure 1a–c), carvacrol (Figure 2a–c), and thymol (Figure 3a–c) were evaluated (the strains were chosen for being multi-resistant and the compounds for presenting the lowest MIC values).

For the eugenol compound in the bacterial population of *S. aureus* 75MR, a bacteriostatic effect was observed at MIC level and a bactericidal effect in MBC at the first hour of exposure. For the populations of *E. coli* 128 MR and *C. albicans*, a bactericidal or fungicidal effect was observed on MIC level at 2 h and 4 h, respectively (Figure 1a–c).

Carvacrol completely inhibited bacterial growth at 4 h for the strain of *E. coli* 128 MR; for *S. aureus* 75MR, the growth inhibition occurred at 2 h, and for the yeast strain of *C. albicans* CUSI, at 4 h (Figure 2a–c).

The compound thymol completely inhibited bacterial growth at the first hour of exposure for *E. coli* 128 MR, while for *S. aureus* 75MR, it was at 4 h, and for the *C. albicans* strain CUSI, the inhibition occurred at 2 h (Figure 3a–c).

As shown in the figures, carvacrol and thymol compounds presented a bactericidal or fungicidal behavior on the bacterial and yeast growth curves at MIC level.

It can be of interest that these compounds are good antimicrobial agents, since at different concentrations, they interrupted the growth of a bacterial population of *E. coli* 128 MR (Gram-negative), *S. aureus* 75MR (Gram-positive), and *C. albicans* CUSI (yeast strain).

Regarding the sensitivity of yeast to the essential oil compound exposure, it was observed that they were more sensitive to carvacrol, presenting the lowest MIC values (0.03–0.06 mg/mL), followed by carvacrol (0.03–0.25 mg/mL), and finally eugenol (0.125–0.25 mg/mL).

## 3. Discussion

The results show that the use of plants and their essential oils used in traditional medicine in the Tehuacan-Cuicatlan valley Puebla can be validated, since several compounds have antimicrobial activity against Gram-positive, Gram-negative bacteria, and yeasts. Several essential oils compounds are lipophilic and can interact with bacterial cytoplasmic membranes, increasing membrane permeability [4,22,23,24,25]. 

The compounds eugenol, carvacrol, and thymol showed the highest antimicrobial activity against Gran positive bacteria, Gran negative bacteria, and yeast. Statistical analysis revealed no significant differences between the compounds and the different groups of microorganisms (bacteria and yeast), indicating that the essential oil compounds can act in different ways. Some authors mention that the sensitivity of Gram-negative bacteria to the different essential oil compounds is due to their outer membrane of lipopolysaccharides, which restricts the diffusion to hydrophobic compounds [15,26,27,28]. 

The structural differences between the compounds are also the cause of the different activities observed. Myrcene (7-methyl-3 -methylene-1,6-octadiene) and ocimene (3,7-dimethyl-1,3,6-octatriene) differ in the position of a double bond (C-3), causing one to present activity on yeast (myrcene) and the other not (ocimeno); carvacrol and thymol differ in the position of a hydroxyl group (in thymol is in ortho position, in carvacrol it is in meta position), causing the deterioration of enzyme systems by binding to the active sites of the enzymes responsible for producing energy and synthesis of structural components, and the destruction or inactivity of genetic material [17,29,30]. Thymol and carvacrol are lipophilic and can enter between the fatty acid chains that make up the lipid bilayers of the membrane, altering the fluidity and permeability of cell membranes; in the case of yeast, compounds such as carvacrol can affect the regulation and the functioning of membrane enzymes that catalyze the synthesis of a series of important polysaccharide components of the cell wall, such as β-glucans, chitin, and mannan, which are involved in cell growth and morphogenesis [14,15].

Gram-positive strains were more sensitive to carvacrol, presenting the lowest MIC values (0.03–0.125 mg/mL), followed by eugenol (0.25–0.75 mg/mL), and lastly thymol (0.25–1.0 mg/mL). Gram-negative bacterial strains were also more sensitive to carvacrol, presenting the lowest MIC values (0.06–0.25 mg/mL), followed by eugenol (0.06–0.5 mg/mL), and lastly thymol (0.25–0.5 mg/mL).

In regards to time-killing curves, it was observed that the effect of eugenol, at MIC level on the populations of *E. coli* 128 MR and *C. albicans* CUSI, showed a bactericidal and fungicidal effect at 2 and 4 h, respectively. Eugenol showed a bactericidal effect against *S. aureus* 75MR after 1 h of contact. Carvacrol and thymol showed a bactericidal and fungicidal effect at MIC level. This behavior is characteristic of a multiple mechanism of action [31].

Although the antimicrobial properties of essential oils and their components have been reviewed in the past, the mechanism of action has not been studied in detail [32]. Components of essential oils such as thymol and carvacrol have previously been reported to have properties that cause membrane disruption in *E. coli* and *S. typhimurium* [12,33]. On the other hand, it was shown that eugenol can disintegrate in the membrane and increase its permeability, and thus causes the death of the organism [4,34]. 

One of the main contributions of this work was to verify the antimicrobial activity of some essential oils compounds present in plants used in traditional medicine, validating their use in the treatment of diseases of possible infectious origin. 

The determination of the specific antimicrobial action of essential oil compounds is important to define possible synergistic activities against resistant microorganisms. In addition, the determination of the specific antimicrobial activity of essential oil compounds is important to define possible synergistic activities against resistant strains, as demonstrated with the thyme essential oil compounds [35,36,37]. 

## 4. Materials and Methods

### 4.1. Essential Oils Compound 

The compounds: α-pinene 98% (# cat. 147524), β-pinene 99% (# cat. 112089), carvacrol 98% (# cat. 282197), eugenol 99% (# cat. E51791), limonene 97% (# cat. 183164), myrcene 90% (# cat. M100005), ocimene 90% (cat. # W353901-1006), cineole 99% (cat. # C80601), methyl salicylate 98% (cat. # W274518), farnesene 90% (cat. # W383902), and thymol 98.5% (cat. # T0501) were obtained from Sigma-Aldrich (Toluca, Mexico).

### 4.2. Microbial Strains 

The following strains of bacteria were used: *Enterobacter agglomerans* ATCC 27155, *Enterococcus faecalis* ATCC 29212, *Escherichia coli* ATCC 25922, *E. coli* ATCC 53218, *E. coli* 10, 28, 128, and 1249 MR multi-resistant strains isolated from clinical cases (donated by the clinical analysis laboratory of the CUSI of the FES Iztacala, UNAM). *Pseudomonas aeruginosa* ATCC 27853, *Salmonella typhi* ATCC 19430, *Staphylococcus aureus* ATCC 12398, *S. aureus* ATCC 29213 (donated by the Microbiology Laboratory of FES-Cuautitlán), *S. aureus* 48, 75, and 83 MR multiresistant strains isolated from clinical cases (donated by the clinical analysis laboratory of the CUSI of the FES Iztacala, UNAM). *Bacillus subtillis, Enterobacter aerogenes, S. epidermidis, Yersinia enterocolitica* (donated by the Clinical Analysis Laboratory of FES Iztacala), *Y. enterocolitica* (isolated from a clinical case and donated by Hospital Angeles Metropolitano), *Proteus mirabilis, Serratia marcences, Streptococcus pneumoniae*, *Vibrio cholerae* (isolated from a clinical case), *V. cholerae* INDRE 206 (isolated from polluted water), and *V. cholerae* (clinical strain pertaining to 01 group, Inaba serotype, El Tor biotype, and enterotoxin producer). These strains were maintained at 4 °C in Mueller Hinton agar (Bioxon), submitted to sensitivity tests (multidiscs Bigaux) and were subcultured twice prior the bioassays. The yeasts tested were: *Candida albicans* ATCC 10231, *C. albicans* ATCC 14065, *C. albicans* isolated from a clinical case (donated by the Clinical Analysis Laboratory of FES Iztacala), *C. albicans, C. glabrata*, and *C. tropicalis* (isolated from a clinical case and donated by Hospital Angeles Metropolitano). The stock culture was maintained on potato dextrose agar (PDA) and subcultured twice prior the bioassays. 

### 4.3. Antibacterial Activity 

The antibacterial activity was measured by the disc diffusion method [38,39]. The microorganisms were grown overnight at 37 °C in 10 mL of Mueller Hinton Broth (Bioxon). The cultures were adjusted with sterile saline solution to obtain turbidity comparable to that of McFarland no. 0.5 standard (10^8^ CFU/mL). Petri dishes containing Mueller Hinton agar (Bioxon) were inoculated with these microbial suspensions. Discs of filter paper (Whatman no. 5) of 5-mm diameter were impregnated with 4 µL of each compound (α-pinene 3.4 mg, β-pinene 3.4 mg, carvacrol 3.9 mg, eugenol 4.3 mg, limonene 3.4 mg, myrcene 3.2 mg, ocimene 3.3 mg, cineole 3.7 mg, methyl salicylate 4.7 mg, farnesene 3.4 mg) and placed on the agar surface (for thymol, a solution was prepared at a concentration of 0.980 g/mL, discs were impregnated with 4 µL = 3.9 mg). Discs impregnated with chloramphenicol (25 µg) were used as positive controls. The plates were incubated overnight at 37 °C and the resulting inhibition zones were measured with a vernier. Each experiment was performed in triplicate. The estimation of the minimal inhibitory concentration (MIC) was carried out by the broth dilution method [40,41]. Ten dilutions of each compound (0.03–4.0 mg/mL) in broth were evaluated. The tubes were inoculated with 10^6^ CFU/mL microorganism suspensions. MIC values were taken as the lowest essential oil concentration that prevents visible bacterial growth after 24 h of incubation at 37 °C and MBC as the lowest concentration that completely inhibited bacterial growth. Chloramphenicol was used as reference standard and compound-free plates as controls. Each experiment was repeated at least three times. The time-killing curve assay was performed using appropriate concentrations of the compounds (corresponding to ½MIC, MIC, and MBC) in 10 mL of broth. One control tube was prepared without compound. Each tube was inoculated with 0.1 mL of a microbial suspension (10^6^ CFU/mL) and incubated at 37 °C for 24 h. Of each tube, 50 µL aliquots were taken in ranges of 2 h. Aliquots were plated on Muller–Hinton agar and incubated for 24 h at 37 °C. Finally, the number of CFU was determined. Death kinetics was expressed in log10 reduction time kill plots [42]. 

### 4.4. Antifungal Activity 

The evaluation of antifungal activity in yeast for each compound was evaluated following the same methodology as for the antibacterial test, but with potato dextrose agar and nystatin as a positive control [40].

### 4.5. Statistics

All experiments were performed in triplicate. The mean and standard deviation of the three experiments were determined. Statistical analysis of the differences between mean values obtained for experimental groups was done by an analysis of variance (ANOVA multifactorial model), where *p*-values of 0.05 or less were considered statistically significant [43]. 

## 5. Conclusions

There are no significant differences in the antimicrobial activity of the compounds on the different bacterial groups and yeasts. The compounds carvacrol, eugenol, and thymol were those that showed the highest antimicrobial activity. Carvacrol was the component with the highest antibacterial activity, followed by eugenol and thymol. Carvacrol was the component with the highest antifungal activity, followed by thymol and lastly eugenol. The compounds ocimene, cineole, and farnesene did not show activity on any yeast strain. A bactericidal effect was observed at concentrations equal to or greater than MIC for eugenol, thymol, and carvacrol. Finally, this study shows that the essential oils of plants used by the population of Tehuacán-Cuicatlán valley share compounds and some of them have antibacterial and fungicidal activity.

## Figures and Tables

**Table 1:** Antimicrobial activity of some compounds in Gram-positive strains.

Strain	Positive Control	α-pinene	β-pinene	Eugenol	Myrcene	Ocimene	Limonene	Carvacrol	Cineole	Methyl Salicylate	Farnesene	Thymol
*B. s* FES-C	16.0 ± 0.5	9.6 ± 1.8	na	16.3 ± 1.5	na	na	8.6 ± 0.5	»	9.0 ± 1.0	na	na	na
*B. s* cc	29.3 ± 2.6	na	na	11.3 ± 0.5	na	11.0 ± 1.0	na	35.6 ± 0.0	na	na	na	na
*E. f*	21.7 ± 1.7	na	na	14.6 ± 1.5	9.0 ± 1.0	10.0 ± 1.7	11.3 ± 0.5	24.3 ± 0.0	8.3 ± 0.5	11.0 ± 0.0	8.6 ± 2.3	»
*M. l*	23.0 ± 0.5	na	na	na	na	na	na	15.0 ± 0.0	na	na	na	na
*S. a* ATCC 12398	28.0 ± 1.6	na	na	11.0 ± 0.0	na	na	10.3 ± 1.1	32.0 ± 0.0	10.0 ± 3.0	16.3 ± 0.9	13.3 ± 1.5	31.6 ± 1.2
*S. a* ATCC 29213	27.6 ± 0.1	na	na	na	na	na	13.6 ± 0.5	27.6 ± 0.0	na	na	7.0 ± 1.0	30.3 ± 0.4
*S. a* 48 MR	22.0 ± 0.0	na	8.0 ± 2.3	18.6 ± 2.0	na	na	na	»	na	10.3 ± 0.4	8.0 ± 0.0	na
*S. a* 75 MR	20.0 ± 0.0	na	5.0 ± 1.4	9.6 ± 1.1	na	na	10.3 ± 1.5	23.3 ± 0.0	na	na	9.0 ± 1.0	26.6 ± 1.2
*S. a* 83 MR	22.0 ± 0.0	na	na	13.0 ± 0.0	na	na	9.6 ± 1.5	26.0 ± 0.0	na	11.6 ± 0.4	9.0 ± 0.0	26.6 ± 1.2
*S. a* cc	14.0 ± 0.0	na	na	12.0 ± 1.0	na	7.3 ± 0.5	na	17.0 ± 0.0	na	14.3 ± 1.6	9.6 ± 0.5	26.3 ± 1.2
*S. e* FES-C	6.7 ± 1.2	na	na	15.3 ± 0.5	na	na	10.3 ± 0.5	na	na	8.0 ± 0.8	na	na

IZ: Inhibition zones (mm). Each bioassay was performed in triplicate ± S. E. (*n* = 3). Positive control: Cloramfenicol 25 µg. *B. s FES-C*: *Bacillus subtilis* FES Cuautitlan, *B. s* cc: *Bacillus subtilis* clinical case, *E. f: Enterococcus feacalis*, *M. l: Micrococcus luteus*, *S. a: Staphylococcus aureus* ATTCC 12398, *S. a: Staphylococcus aureus* ATTCC 29213, *S. a* 48 MR: *Staphylococcus aureus* 48 multi-resistant, *S. a* 75 MR: *Staphylococcus aureus* 75 multi resistant, *S. a* 83 MR: *Staphylococcus aureus* 83 multi resistant, *S. a* cc: *Staphylococcus aureus* clinical case. *S. e: Staphylococcus epidermidis* FES Cuautitlan, na: no activity.

**Table 2:** Antimicrobial activity of some compounds in Gram-negative strains.

Strain	Positive Control	α-pinene	β-pinene	Eugenol	Myrcene	Ocimene	Limonene	Carvacrol	Cineole	Methyl Salicylate	Farnesene	Thymol
*E. ae* FES-C	12.0 ± 0.5	na	na	13.3 ± 0.5	na	9.0 ± 0.0	16.3 ± 2.8	26.3 ± 1.1	na	na	8.6 ± 0.5	29.6 ± 0.4
*E. ae* cc	19.3 ± 0.5	na	8.0 ± 3.5	na	na	na	na	33.0 ± 1.0	na	na	na	na
*E. c* 10 MR	22.0 ± 0.0	na	na	15.3 ± 1.5	na	na	7.0 ± 0.0	17.6 ± 0.5	8.6 ± 1.1	9.3 ± 0.9	9.6 ± 0.5	na
*E. c* 1249 MR	23.0 ± 0.0	na	na	14.3 ± 0.5	na	na	na	24.3 ± 1.1	na	na	na	28.0 ± 1.6
*E. c* 128 MR	25.0 ± 0.0	na	na	na	na	na	8.3 ± 0.5	27.3 ± 0.5	na	na	na	18.6 ± 0.9
*E. c* 28 MR	24.0 ± 0.0	na	na	20.0 ± 2.0	na	na	10.0 ± 0.0	na	na	na	na	na
*E. c* ATCC 53228	21.7 ± 1.7	11.6 ± 2.0	na	15.3 ± 0.5	8.3 ± 2.5	9.6 ± 1.5	25.6 ± 1.1	30.0 ± 0.5	na	na	na	»
*E. c* cc	9.0 ± 0.0	na	na	na	na	na	9.6 ± 0.5	23.6 ± 0.5	7.5 ± 0.7	na	na	25.0 ± 3.5
*P. ae*	7.3 ± 0.6	na	na	12.3 ± 1.1	na	8.0 ± 2.0	na	28.6 ± 1.1	8.0 ± 0.0	8.0 ± 0.8	7.6 ± 0.5	28.6 ± 1.2
*P. m*	13.3 ± 0.5	na	na	14.6 ± 2.0	na	na	na	20.3 ± 0.5	9.6 ± 0.5	10.6 ± 0.4	na	»
*S. t*	25.7 ± 0.5	na	na	20.0 ± 1.0	na	na	7.6 ± 0.5	26.6 ± 1.5	na	na	na	33.3 ± 1.2
*V. ch* Tor	6.7 ± 0.6	na	na	12.6 ± 1.1	na	9.6 ± 1.5	na	29.0 ± 1.7	na	8.3 ± 1.24	8.3 ± 0.5	»
*V. ch* Agua	10.0 ± 1.0	na	na	13.3 ± 1.1	8.0 ± 0.0	7.0 ± 0.0	10.6 ± 0.5	22.3 ± 0.5	na	7.3 ± 0.4	na	32.3 ± 1.2
*V. ch* cc	27.7 ± 0.5	na	na	13.6 ± 0.5	na	6.6 ± 1.5	13.0 ± 1.0	25.0 ± 0.0	na	8.6 ± 0.9	8.3 ± 0.5	30.3 ± 0.4
*Y. e* CUSI	8.3 ± 0.1	na	na	18.6 ± 5.5	na	na	12.3 ± 0.5	na	10.3 ± 0.5	na	na	»
*Y. e* HA	25.7 ± 0.5	na	na	24.0 ± 1.0	10.3 ± 2.0	10.3 ± 1.5	9.3 ± 1.1	24.3 ± 0.5	na	na	10.0 ± 0.0	»

IZ: Inhibition zones (mm). Each bioassay was performed in triplicate ± S. E. (*n* = 3). Positive control: Cloramfenicol 25 µg. *E. ae FES-C: Enterobacter aerogenes*, *E. ae cc: Enterobacter aerogenes clinical case*, *E. c* MR: *Escherichia coli* multiresistant strains, *E. c: Escherichia coli* ATCC 53218, *E. c cc*: *Escherichia coli* clinical case, *P. ae: Pseudomonas aeruginosa* ATCC 27853, *P. m*: *Proteus mirabilis, S. t: Salmonella* *tiphymurium* ATCC 19430, *Vch* Tor: *Vibrio cholerae* CDC V12, *V. ch* Agua: *Vibrio cholerae* INDRE 206, *Vch* cc: *Vibrio cholerae* clinical case, *Y. e* CUSI: *Yersinia enterocolitica*. *Ye* HA: *Yersinia enterocolitica* clinical case, »: total growth inhibition, na: no activity.

**Table 3:** Antimicrobial activity of some compounds in yeast strains.

Strain	Positive Control	α-pinene	β-pinene	Eugenol	Myrcene	Limonene	Carvacrol	Methyl Salicylate	Thymol
*C a.* ATCC 14065	10.0 ± 0.0	12.0 ± 0.8	na	30.3 ± 1.5	12.0 ± 1.7	15.3 ± 1.5	32.3 ± 2.0	»	»
*C a.* CUSI	10.0 ± 0.0	na	7.7 ± 0.9	20.0 ± 1.0	10.3 ± 1.5	14.0 ± 2.6	39.0 ± 1.7	»	»
*C a.*cc	10.0 ± 0.0	14.3 ± 2.4	9.3 ± 1.8	20.6 ± 1.5	11.3 ± 1.5	21.0 ± 7.81	»	na	»
*C. glabrata*	8.0 ± 0.0	na	10.0 ± 0.0	14.3 ± 1.1	9.3 ± 1.5	14.6 ± 0.5	34.6 ± 4.0	»	»
*C. tropicalis*	9.0 ± 0.0	na	11.0 ± 1.4	16.0 ± 0.0	10.6 ± 1.1	16.6 ± 3.5	34.0 ± 6.0	na	»
*C. tropicalis* cc	9.0 ± 0.0	9.3 ± 1.2	na	13.0 ± 2.0	10.6 ± 0.5	13.6 ± 0.5	»	»	»
*C a.* 17 MR	10.0 ± 0.0	na	11.3 ± 1.4	18.3 ± 3.0	10.0 ± 1.0	8.3 ± 7.23	»	na	»
*C a.* 18 MR	10.0 ± 0.0	12.3 ± 1.4	na	16.3 ± 3.0	11.3 ± 0.5	7.6 ± 6.6	»	na	»

IZ: Inhibition zones (mm). Each bioassay was performed in triplicate ± S. E, (*n* = 3). Positive control: Nistatine 30 µg. *C a*. ATCC 14065, *C. albicans* ATCC 14065, *C a.* CUSI: *Candida albicans* clinical case, *C a.* cc: *Candida albicans* clinical case, *C. glabrata, C. tropicalis* clinical case, *C. a* 17 and 18 MR: *C. albicans* 17 and 18 multiresistant strains, »: total growth inhibition, na: no activity.

**Table 4:** Minimal inhibitory concentrations of some compounds in Gram-positive strains.

Strain	Positive Control	α-pinene	β-pinene	Eugenol	Myrcene	Ocimene	Limonene	Carvacrol	Cineole	Methyl salicylate	Farnesene	Thymol
*B. s* FES-C	0.002	>4.00	nd	0.25	nd	nd	2.00	0.125	1.00	nd	nd	nd
*B. s* cc	0.002	nd	nd	0.25	nd	nd	nd	0.125	nd	nd	nd	nd
*E. f*	0.003	nd	nd	0.75	4.00	4.00	2.00	0.030	1.00	>2.00	nd	nd
*M. l*	0.003	nd	nd	nd	nd	nd	nd	0.125	nd	nd	nd	nd
*S. a* ATCC 12398	0.001	nd	nd	0.50	nd	nd	2.00	0.125	1.00	>2.00	nd	0.25
*S. a* ATCC 29213	0.008	nd	nd	nd	nd	nd	1.50	0.125	nd	nd	>2.00	1.00
*S. a* 48 MR	0.007	nd	nd	0.50	nd	nd	nd	0.062	nd	nd	nd	nd
*S. a* 75 MR	0.007	nd	>4.00	0.50	nd	nd	nd	0.125	nd	nd	>2.00	0.50
*S. a* 83 MR	0.007	nd	nd	0.50	nd	nd	nd	0.125	nd	>2.00	>2.00	nd
*S. a* cc	0.002	nd	nd	0.50	nd	1.50	nd	0.125	nd	>2.00	>2.00	nd
*S. e* FES-C	0.002	nd	nd	0.50	nd	nd	2.00	nd	nd	nd	nd	0.50

MIC (mg/mL). Each bioassay was performed in triplicate ± S. E. (*n* = 3). Positive control: Cloramfenicol 25 µg. *B. s FES-C*: *Bacillus subtilis* FES Cuautitlan, *B. s* cc: *Bacillus subtilis* clinical case, *E. f: Enterococcus feacalis*, *M. l: Micrococcus luteus*, *S. a: Staphylococcus aureus* ATTCC 12398, *S. a: Staphylococcus aureus* ATTCC 29213, *S. a* 48 MR: *Staphylococcus aureus* 48 multi-resistant, *S. a* 75 MR: *Staphylococcus aureus* 75 multi resistant, *S. a* 83 MR: *Staphylococcus aureus* 83 multi resistant, *S. a* cc: *Staphylococcus aureus* clinical case. *S. e: Staphylococcus epidermidis* FES Cuautitlan, nd: no determinate.

**Table 5:** Minimal inhibitory concentrations of some compounds in Gram-negative strains.

Strain	Positive Control	α-pinene	β-pinene	Eugenol	Myrcene	Ocimene	Limonene	Carvacrol	Cineole	Methyl salicylate	Farnesene	Thymol
*E. ae* FES-C	0.004	nd	nd	0.50	nd	4.00	nd	0.125	nd	nd	nd	nd
*E. ae* cc	0.004	nd	nd	nd	nd	nd	nd	0.125	nd	nd	nd	nd
*E. c* 10 MR	0.004	nd	nd	0.25	nd	nd	2.00	0.125	nd	>2.00	>2.00	nd
*E. c* 1249 MR	0.004	nd	nd	0.50	nd	nd	nd	0.50	nd	nd	nd	0.25
*E. c* 128 MR	0.004	nd	nd	0.25	nd	nd	nd	0.125	nd	nd	nd	0.25
*E. c* 28 M*R*	0.004	nd	nd	0.50	nd	nd	2.00	nd	nd	nd	nd	1.00
*E. c* ATCC 53228	0.004	0.25	nd	0.25	2.00	nd	0.50	0.125	nd	nd	nd	nd
*E. c* cc	0.004	nd	nd	0.25	nd	nd	2.00	0.25	nd	nd	nd	nd
*P. ae*	0.008	nd	nd	nd	nd	nd	nd	0.125	nd	nd	nd	2.00
*P. m*	0.004	nd	nd	0.50	nd	4.00	nd	0.062	nd	>2.00	nd	nd
*S. t*	0.002	nd	nd	0.50	nd	nd	nd	0.125	nd	nd	nd	0.50
*V. ch* Tor	0.001	nd	nd	0.50	nd	nd	2.00	0.25	nd	>2.00	nd	nd
*V. ch* Agua	0.001	nd	nd	0.50	4.00	4.00	nd	0.25	nd	>2.00	nd	nd
*V. ch* cc	0.001	nd	nd	0.50	nd	4.00	2.00	0.25	nd	>2.00	nd	nd
*Y. e* CUSI	0.004	nd	nd	0.062	4.00	4.00	2.00	nd	0.75	>2.00	>2.00	nd
*Y. e* HA	0.004	nd	nd	0.50	nd	nd	2.00	0.25	nd	nd	nd	nd

MIC (mg/mL). Each bioassay was performed in triplicate ± S. E. (*n* = 3). Positive control: Cloramfenicol 25 µg. *E. ae FES-C: Enterobacter aerogenes*, *E. ae cc: Enterobacter aerogenes clinical case*, *E. c* MR: *Escherichia coli* multiresistant strains, *E. c: Escherichia coli* ATCC 53218, *E. c cc*: *Escherichia coli* clinical case, *P. ae: Pseudomonas aeruginosa* ATCC 27853, *P. m*: *Proteus mirabilis, S. t: Salmonella* *tiphymurium* ATCC 19430, *Vch* Tor: *Vibrio cholerae* CDC V12, *V. ch* Agua: *Vibrio cholerae* INDRE 206, *Vch* cc: *Vibrio cholerae* clinical case, *Y. e* CUSI: *Yersinia enterocolitica*. *Ye* HA: *Yersinia enterocolitica* clinical case, nd: no determinate.

**Table 6:** Minimal inhibitory concentrations of some compounds in yeast strains.

Strain	Positive Control	α-pinene	β-pinene	Eugenol	Myrcene	Limonene	Carvacrol	Methyl salicylate	Thymol
*C a.* ATCC 14065	0.011	0.50	nd	0.25	2.00	2.00	0.03	>2.00	0.125
*C a.* CUSI	0.011	nd	0.50	0.125	2.00	2.00	0.03	>2.00	0.03
*C a.* cc	0.011	0.50	1.50	0.25	2.00	2.00	0.03	nd	0.25
*C. glabrata*	0.008	nd	2.00	0.25	2.00	2.00	0.062	>2.00	0.125
*C. tropicalis*	0.009	nd	4.00	0.25	3.00	2.00	0.03	nd	0.125
*C. tropicalis* cc	0.009	0.50	nd	0.25	1.00	2.00	0.03	nd	0.25
*C a.* 17 MR	0.011	nd	nd	0.25	2.00	2.00	0.03	>2.00	0.125
*C a.* 18 MR	0.011	nd	nd	0.25	4.00	2.00	0.03	nd	0.125

MIC (mg/mL). Each bioassay was performed in triplicate ± S. E, (*n* = 3). Positive control: Nistatine 30 µg. C a ATCC 14065, *C. albicans* ATCC 14065, *Ca* CUSI: *Candida albicans* clinical case, *Ca* cc: *Candida albicans* clinical case, *C. glabrata, C. tropicalis* clinical case, *C. a* 17 and 18 MR: *C. albicans* 17 and 18 multiresistant strains, nd: no determinate.
